# Prevalence and molecular characterization of infectious bronchitis virus isolated from chicken in Bangladesh

**DOI:** 10.14202/vetworld.2019.909-915

**Published:** 2019-06-28

**Authors:** Zafar Ahmed Bhuiyan, Md. Zulfekar Ali, Mohammad Moktader Moula, Md. Giasuddin, Zahed Uddin Mahmood Khan

**Affiliations:** 1 Department of Botany, Jahangirnagar University, Savar, Dhaka 1341, Bangladesh; 2 Animal Health Research Division, Bangladesh Livestock Research Institute, Savar, Dhaka 1341, Bangladesh; 3 Central Poultry Laboratory, Nourish Poultry and Hatchery Ltd., Dhaka, Bangladesh

**Keywords:** chicken, infectious bronchitis virus, isolation, molecular, prevalence

## Abstract

**Aim:**

The present study was aimed to determine the prevalence of infectious bronchitis virus (IBV) as well as virus isolation, identification, and molecular characterization of various strains circulating in Bangladesh.

**Materials and Methods:**

A total of 371 swabs and organ samples were collected from four types of chicken including layer, Sonali (local), broiler, and broiler breeder under eight districts (Rangpur, Bogura, Tangail, Dhaka, Gazipur, Mymensingh, Jamalpur, and Cumilla) during 2014-2016 in Bangladesh.

**Results:**

Out of 371 samples, 65 samples were positive in reverse transcriptase polymerase chain reaction (RT-PCR) for molecular identification of IBV. The overall prevalence was 17.52% recorded and among the selected types of chicken, the highest prevalence of IBV was found in layer that was 42.22% followed by 17.24% in Sonali, 14.93% in broiler breeder, and lowest prevalence was 11.94% in broiler chicken, respectively. Moreover, the prevalence of IBV was recorded highest in aged chicken at 41-60 weeks, which was 54.55% in layer, 27.27% in Sonali, and, afterward, 14.68% was found in broiler breeder, respectively. Frequency of IBV more frequently in winter (22.67%) followed by rainy (15.87%) and summer season (11.58%). The highest prevalence of IBV was found Tangail district (41.67%) followed by Mymensingh (24.42%), Gazipur (19.32%), Dhaka (15.38%), Jamalpur (16.67%), Bogura (13.68%), Cumilla (5.88%), and Rangpur (9.26%), respectively. Samples that were found high positive in IBV RT-PCR (Ct value below 30) were subjected to inoculation into chicken egg embryo to observe characteristic changes in chicken embryo. Swabs and organ samples were processed and passaged in 9-day-old embryonated chicken eggs through allantoic cavity route. IBV virus suspected samples inoculated into chicken egg embryos after 3-5 passages showed dwarfing and curling of the embryos which are characteristic lesions of IBV. Allantoic fluid was collected from all inoculated eggs and performed partial sequencing of S1 gene for three isolates. After sequencing, the phylogenetic tree was constructed from the nucleotide sequences of IBV isolates. Two of the isolates are 4/91 IBV and another one matched with QX-like IBV.

**Conclusion:**

The results revealed that the three isolates from different places in Bangladesh were identified for the 1^st^ time as which will help for IBV control strategy.

## Introduction

The poultry industry in Bangladesh has grown leaps and bound since the past two decades. Poultry farming not only assists in upgrading the financial condition but also makes a substantial contribution to human nutrition [1]. The total contribution of livestock subsectors to gross domestic products in Bangladesh is approximately 1.54% and in agricultural products 13.62% [2]. It also generates 13% of foreign currency and provides 20% fulltime employment and 45% partial employment of rural population [2]. However, it is the matter of regret that poultry industry in Bangladesh continuously facing challenges in several ways. Increased prevalence of disease outbreaks which reemergence of various pathogens with higher virulence due to constant natural evolution of variant strains as a result of recombination of virus and frequent use of live vaccine are the main reasons for such challenges [3,4].

One of the most economical important diseases of poultry is infectious bronchitis virus (IBV) [5]. It is highly contagious and causing devastating economic losses to chickens [6,7]. The virus causes respiratory disease characterized by tracheal rales, coughing, and sneezing along with excess accumulation of mucus in bronchi and reduced growth rate in broilers, nephritis, urolithiasis, and irreparable damage to oviduct, leading to abnormal eggs production with high mortality rates in affected poultry flocks [8,9]. IBV is an enveloped, positive-sense, single-stranded RNA virus, belonging to the genus *Gamma coronavirus* [10]. The spike (S) glycoprotein is cleaved into two smaller proteins, namely S1 (amino-terminal component) and S2 (carboxy-terminal component). The S1 portion has two hypervariable regions which cause virus neutralization and serotype-specific antigenic determinants that are responsible for binding to the host cell, neutralization, and immune response [11] and also important for involving pathogenicity of the virus to the host [12].

IBV can affect chicken of all ages [13]. However, chicks of 2 to 3 weeks of age are extremely susceptible. Mortality rates as high as 40%-90% were observed in affected chicks [14,15]. The disease is transmitted through the air-borne, mechanical transmission between birds, houses, and farms. Air-borne transmission mainly occurs through aerosol and mechanical transmission occurs by personal contact, material, and equipment sharing in between farm and flock. Confirmatory diagnosis of IB can be achieved by a number of tests including agar gel precipitation test, hemagglutination inhibition, and enzyme-linked immune sorbent assay. The more traditional methods used to determine the serotype of the virus include virus isolation and neutralization tests which are being fast replaced with rapid, specific, and confirmatory tests such as reverse transcriptase polymerase chain reaction (RT-PCR) and restriction fragment length polymorphism [16]. The virus isolation is usually performed in specific pathogen free (SPF) 9-11-day-old embryonated chicken eggs (ECE) [17]. RT-PCR has been used mostly for determining field strain of IBV. The antigenic variation of spike protein, especially S1 subunit protein, is determined by nucleotide sequencing method [7].

The present study was aimed to determine the prevalence of IBV as well as virus isolation, identification, and molecular characterization of various strains circulating in Bangladesh.

## Materials and Methods

### Ethical approval

The Animal Ethics Committee of Bangladesh Livestock Research Institute approved the study.

### Sample collection and preparation

Oropharyngeal, tracheal, and cloacal swab samples were collected in virus transport media containing falcon tube and organ samples (trachea, lungs, and kidney) which were collected in sterilized zipper-lock bag from the breeder, broiler, layer, and Sonali (local) chicken that was showing clinical respiratory sign from each sampling area. The chickens were vaccinated with IBV vaccine against MA5, H120, and IB 4/91 strain according to standard vaccine schedule from a commercial source. A total number of 371 swab and organ samples were collected from the respective farm. Among them, broiler breeder (n=134), layer (n=45), Sonali (n=58), and broiler (n=134) were considered for sampling. After collection, the sample transported in ice box and stored at −20°C refrigerator in the laboratory. The organ samples were taken into sterile pestle and homogenized with the help of sterile scissors and mortar and added 20% phosphate-buffered saline to make a suspension and then centrifuged at 1000 rpm for 10 min. Supernatant fluid was taken by syringe for RNA extraction. Swab samples were homogenized by vortexing at 2-3 s and selected for RNA extraction.

### RNA extraction for RT-PCR

RNA extraction was done with Purelink RNA Mini Kit, P/N: 12183018A by Invitrogen, USA, as per manufacturer’s instructions.

### Real-time RT-PCR for IBV detection

The one-step RT-PCR was performed using S1 gene-specific primer and probe with thermal condition described by Callison *et al*. [18] (Tables-1 and 2). RT-PCR of the extracted RNA was conducted using the commercially available AgPath-ID One-Step RT-PCR kit (Invitrogen, USA). Thermocycler used for PCR was Applied Biosystem 7500 by Life Technologies.

**Table-1:** IBV primer and probe details.

Name	Sequence (5’ to 3’)	Nucleotide positions	Reference
IBV5 GU391	5’- GCT TTT GAG CCT AGC GTT-3’	391-408	[18]
IBV5 GL533	5’- GCC ATG TTG TCA CTG TCT ATT G-3’	533-512	
IBV5 G probe	5’- FAMCAC CAC CAG AAC CTG TCA CCT C-BHQ1-3’	494-473	

IBV=Infectious bronchitis virus

**Table-2:** Master mix composition and RT-PCR condition.

S. No.	Reagent	Volume (µl)	Thermal profile
1	2×-RT-PCR buffer	12.5	50°C for 30 min; 94°C for 15 min; 40 cycles of 94°C for 15 s followed by 60°C for 60 s
2	25×RT-enzyme	1.0	
3	Forward primer	1.25		
4	Reverse primer	1.25		
5	Probe	0.25		
6	Nuclease-free water	3.75		
7	Template RNA	5.0		
	Total	25.0		

RT-PCR=Reverse transcriptase polymerase chain reaction

### Propagation and isolation of virus

Samples that were found high positive in IBV RT-PCR (Ct ≤30) were subjected to inoculate at the 9^th^ day SPF chicken egg embryo through allantoic cavity route and observe curling and dwarfing [7] in chicken embryo according to the OIE guideline [15]. After 48 h of inoculation, eggs were chilled at 4°C for at least 6 h and allantoic fluid was harvested.

### Sequences and phylogenetic analysis

**Figure-1:**
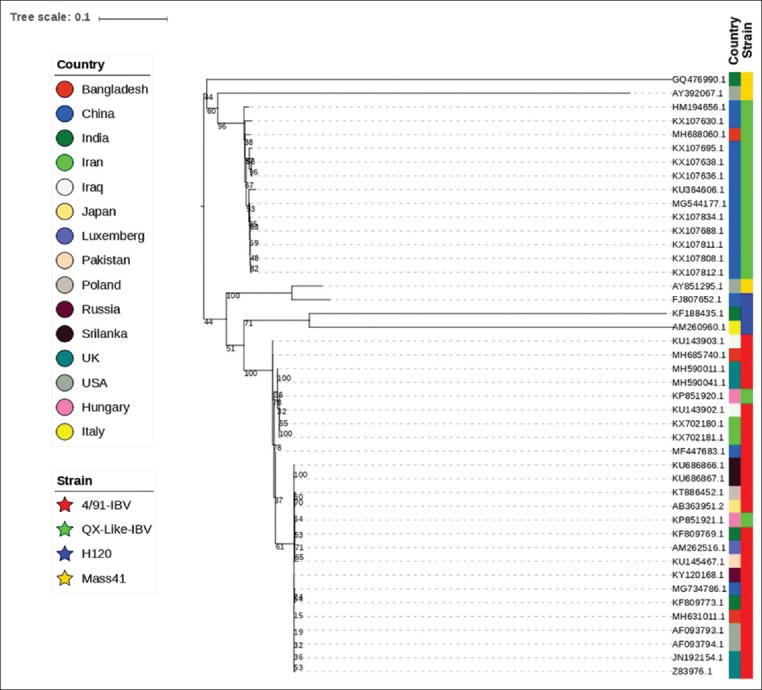
Phylogenetic tree of amino acid sequences of S1 genes of isolated infectious bronchitis virus. The isolates used in this study indicated by red color.

Allantoic fluid in FTA card was sent to the CEVA Phylexia Laboratory, Hungary, for Sanger sequencing of IBV. Sequences of the S1 gene of the Bangladeshi IBV isolates were compared with published IBV sequences deposited in the GenBank database using a BLAST. Sequence identities by BLAST analysis were included in alignment and phylogenetic construction. A phylogenetic tree of the nucleotide sequences was constructed using R version 3.4.0 and under package phangorn version 2.4.0, and package ape version 5.1 (R Core Team, USA) [19] (Figure-1).

## Results

A total of 371 samples were collected and tested for IBV by the real-time RT-PCR test. The overall prevalence of IBV was found to be 17.52% (65/371). Among the selected types of chicken, the highest prevalence of IBV was found in commercial layer chicken that was 42.22% (19/45) and the lowest prevalence was showed broiler chicken as 11.94% (16/134). The prevalence of 17.24% (10/58) and 14.93% (20/134) was showed in Sonali and broiler breeder chicken, respectively (Table-3).

**Table-3:** Prevalence of infectious bronchitis virus according to the type of poultry.

S. No.	Type	Total sample tested	Results		

			Positive	Negative	Prevalence (%)
1	Commercial layer	45	19	26	42.22
2	Sonali	58	10	48	17.24
3	Broiler	134	16	118	11.94
4	Broiler breeder	134	20	114	14.93
Total		371	65	306	17.52

According to the age of the chicken in both commercial layer and Sonali chicken, the highest prevalence was found in age between 41 and 60 weeks, which was 54.55% (6/11) and 27.27% (3/11), respectively. However, in case of broiler breeder chicken, it was highest at 21-40 weeks of age than 41-60 weeks of age, which was 16% (4/25) and 14.68% (16/109), respectively. On the other hand, in broiler chicken, age divided into two groups due to short life cycle and found prevalence 12.5% (1/8) at 0-16 days of age and prevalence 11.9% (15/126) at 17-32 days of age (Table-4).

**Table-4:** Prevalence of infectious bronchitis virus according to the age of poultry.

Type	Age	Total sample tested	Results	

			Positive sample	Prevalence (%)
Commercial layer	0-20 weeks	9	2	22.22
	21-40 weeks	12	4	33.33
	41-60 weeks	11	6	54.55
	61-80 weeks	13	7	53.85
		45	19	42.22
Sonali	0-20 weeks	6	1	16.67
	21-40 weeks	31	5	16.13
	41-60 weeks	11	3	27.27
	61-80 weeks	10	1	10.00
		58	10	17.24
Broiler	0-16 days	8	1	12.50
	17-32 days	126	15	11.90
		134	16	11.94
Broiler breeder	21-40 weeks	25	4	16.00
	41-60 weeks	109	16	14.68
		134	20	12.94
Total		371	65	17.52

Three years (2014-2016) were observed during the study period, among three seasons, highest 22.67 (34/150) and lowest 11.58% (11/95) prevalence was found in winter and summer season correspondingly. In rainy season, 15.87% (20/126) prevalence was found (Table-5).

**Table-5:** Prevalence of infectious bronchitis virus according to the season of poultry.

Season	Month	Total sample tested	Results	

			Positive sample	Prevalence (%)
Summer	March	28	6	21.43
	April	54	4	7.41
	May	13	1	7.69
		95	11	11.58
Rainy	June	22	4	18.18
	July	18	8	44.44
	August	13	6	46.15
	September	34	1	2.94
	October	39	1	2.56
		126	20	15.87
Winter	November	14	4	28.57
	December	72	16	22.22
	January	31	5	16.13
	February	33	9	27.27
		150	34	22.67
Total		371	65	17.52

Table-6 shows the prevalence of IBV in eight different districts over Bangladesh, where 371 samples were tested and found the highest prevalence in Tangail district. It was 41.67% (5/12) chickens ware affected. The lowest prevalence was found in Cumilla district as 5.88% (1/17). The prevalence of another six districts was 9.26% (5/54), 13.68% (13/95), 15.38% (2/13), 19.32% (17/88), 24.42% (21/86), and 16.67% (1/6) of Rangpur, Bogura, Dhaka, Gazipur, Mymensingh, and Jamalpur, respectively.

**Table-6:** Prevalence of infectious bronchitis virus according to the region of poultry.

S. No.	Region	Total sample tested	Results	

			Positive sample	Prevalence (%)
1	Rangpur	54	5	9.26
2	Bogura	95	13	13.68
3	Tangail	12	5	41.67
4	Dhaka	13	2	15.38
5	Gazipur	88	17	19.32
6	Mymensingh	86	21	24.42
7	Jamalpur	6	1	16.67
8	Cumilla	17	1	5.88
Total		371	65	17.52

The identified IBV samples were inoculated into 9-day-old SPF ECE through chorioallantoic membrane route. The chicken embryos were shown characteristic lesions of growth such as curled and dwarfed embryos with covered by a thickened amnion after the third passage propagation. Then, IBV was isolated and reconfirmed by RT-PCR.

The partial nucleotide sequence analysis of S1 glycoprotein gene was performed in the isolate CK/BD/IBV/NPHL1, CK/BD/IBV/NPHL2, and CK/BD/IBV/NPHL3 and found further defined genetic relationship with two IBV subtypes; one isolate was QX-like IBV and another two isolates were 4/91 IBV circulating in the selected regions, shown in phylogenetic tree (Figure-1). The sequenced nucleotides were submitted into GenBank under accession number MH631011.1 (isolate: CK/BD/IBV/NPHL1), MH688060.1 (isolate: CK/BD/IBV/NPHL2), and MH685740.1 (isolate: CK/BD/IBV/NPHL3).

The phylogenetic analysis demonstrated that the isolates of QX-like IBV are 99% related with isolated of China (accession no. KX107695.1) and two isolates of IBV 4/91 were related with Indian isolates (KF809773.1) and Poland isolates (KT886452.1). The results indicate that both QX-like IBV and 4/91 strains are circulating in chickens in Bangladesh.

## Discussion

In this study, the prevalence and characterization of IBV in between 2014 and 2016 were identified. The prevalence of IBV was widespread in the selected areas of Bangladesh and overall 17.52% prevalence was found by identification of S1 gene in quantitative PCR (qPCR). Overall, 58% seroprevalence of IBV were demonstrated by Barua *et al*. [20]. Another researcher Das *et al*. [21] finds that a total of 79.38% sera samples of chickens of selected four districts of Bangladesh were seropositive. Although they do not differentiate the seroprevalence of IBV between vaccinated and non-vaccinated chickens. In Pakistan, overall seroprevalence of IBV was 88% reported by Ahmed *et al*. [22]. They represent the antibody status by enzyme-linked immunosorbent assay test procedure that is quite different from molecular prevalence by qPCR tests.

Of three types of chicken, commercial layer chickens were the highest (42.22%) prevalence of IBV and lowest prevalence was in broiler chickens. For other two types, it was 17.24% in Sonali and 14.93% in broiler breeder chicken. Except broiler, the other three types of chickens reared for more than 60 weeks of age that increased the chances of harbor of IBV infection. Jackwood [23] and Seger *et al*. [24] reported that the prevalence of IBV in layer is 74% in Iraq by RT-PCR, whereas 60% infection carried by 793/B strain of IBV.

The prevalence of IBV had shown a relation in age of chicken. The highest prevalence of IBV was found in the age between 41 and 60 weeks of age of commercial layer and Sonali chicken. On the other hand, it was highest at 21-40 weeks of age in broiler breeder and at 0-16 days of age in broiler.

Javed *et al*. [14] demonstrated that the IBV is more prevalent in the layer chicken at age >40 weeks of age and in broiler chicken at age >7 days in Pakistan. However, mortality increased in young chicken than older due to respiratory distress and kidney manifestation [7] and it may reach up to 20-30% [25,26].

In the present study, the prevalence of IBV becomes highest during winter season (November-February) and it was 22.67%. Researchers shown that the incidence of IBV increased during winter season due to cold weather is more favorable to IBV for survival and spread through droplets and dust [14,23,27].

The samples for this study were collected from eight districts of Bangladesh, in which Tangail district revealed the highest prevalence of IBV and it was 33.30% and the second highest prevalence found in Mymensingh district as 24.42%. It has been mentioned by Sylvester *et al*. [28], that the transmission and severity of IBV are correlated with flock density. The Tangail and Mymensingh might be the high density of poultry flocks that increased the rates of the prevalence of IBV [29].

Genetically, the S1 gene sequenced data identified that the two serotypes of IBV were circulating during the period of 2014-2016 that is QX-like IBV and 4/91 strains. The comparative analysis S1 gene hypervariable region of IBV in nucleotide sequence is a strong tool for the identification of circulating field variants of IBV [16,30,31].

## Conclusion

The study focused on the overview of the prevalence and molecular characterization of IBV circulating in Bangladesh. The overall 17.52% prevalence with highest in commercial layer chickens comparatively Sonali, broiler, and broiler breeder chickens. The age of chicken over 41 weeks shows more prevalent. The prevalence of IBV ubiquitous round the year but increase prevalence during winter season. According to location, it was highly prevalent in Tangail district. Finally, the sequencing and phylogeny analysis of S1 gene of IBV demonstrated the QX-like IBV and 4/91 strain of IBV were circulating in the environment.

## Authors’ Contributions

ZAB, MZA, MG, and ZUMK: Conceived and designed the study. ZAB, MZA, and MMM: Collected samples and performed the experiments and laboratory analyses. MZA: Performed the data analyses. ZAB and MZA: Drafted the manuscript. MMM, MG, and ZUMK: Revised the manuscript critically. All authors read and approved the final manuscript.
